# Radiosensitization of NET cells by HSP90 inhibitor ganetespib is mediated through pleiotropic stress responses

**DOI:** 10.1186/s13550-025-01346-z

**Published:** 2025-11-21

**Authors:** Pleun A.M. Engbers, Thom G.A. Reuvers, José María Heredia-Genestar, Jiang Chang, Nicole S. Verkaik, Mariangela Sabatella, Julie Nonnekens

**Affiliations:** 1 Department of Molecular Genetics, Erasmus MC Cancer Institute, Erasmus University Medical Center , Rotterdam, The Netherlands; 2 Department of Radiology and Nuclear Medicine, Erasmus MC Cancer Institute, Erasmus University Medical Center , Rotterdam, The Netherlands; 3 Erasmus University Medical Center, Dr. Molewaterplein 40, 3015GD, Rotterdam, The Netherlands

**Keywords:** Peptide receptor radionuclide therapy, ^177^Lu-DOTA-TATE, HSP90, Ganetespib, Radiosensitization, Neuroendocrine tumors

## Abstract

**Background:**

Peptide receptor radionuclide therapy (PRRT) employing [^177^Lu]Lu-[DOTA-Tyr^3^]octreotate has been established as treatment for patients with metastatic neuroendocrine tumors (NETs) that overexpress the somatostatin receptor (SSTR). While PRRT improves survival and quality of life, curative responses remain rare. One way to enhance PRRT efficacy is to combine it with radiosensitizing agents such as heat shock protein 90 (HSP90) inhibitors. HSP90 is a highly conserved molecular chaperone essential for the maturation and stabilization of over a hundred proteins, including proteins involved in the DNA damage response and oncogenic signaling. HSP90-inhibition has been shown to potentiate PRRT, however the mechanism behind this radiosensitizing effect remains unknown. This study aimed to elucidate mechanisms involved in the radiosensitizing effect of HSP90 inhibition.

**Results:**

The radiosensitizing effect of HSP90 inhibitor ganetespib in the context of PRRT and external beam radiotherapy (EBRT) was tested using viability assays for NET cell models GOT1 and BON1-SSTR2. Ganetespib significantly enhanced radiation-induced cytotoxicity in both models. To explore underlying mechanisms, we assessed DNA double-strand break (DSB) repair by quantifying 53BP1 foci numbers, and functionally evaluated DSB repair pathways by RAD51 foci quantification and end-joining assay. Although HSP90 inhibition reduced RAD51 foci numbers, its effect on non-homologous end joining and overall DSB persistence was limited. Finally, potential DSB repair-independent mechanisms of radiosensitization were assessed for GOT1 cells using RNA sequencing. Transcriptomic analysis revealed enrichment of pathways related to loss of HSP90 function, such as protein folding and response to heat stress, following combination treatment. This was consistent with effects observed after HSP90 inhibitor monotherapy.

**Conclusions:**

Given the lack of significant effects on direct DNA repair or transcriptomic responses, our findings suggest that HSP90 inhibition radiosensitizes NET cells by inducing a pleiotropic effect on multiple stress-related pathways at the protein level, rather than solely through disruption of DNA damage response mechanisms. This effect is likely driven by loss of HSP90 function and subsequent cumulated unfolded protein and proteotoxic stress.

**Supplementary Information:**

The online version contains supplementary material available at 10.1186/s13550-025-01346-z.

## Background

Peptide receptor radionuclide therapy (PRRT) has been established as an effective treatment for patients with metastatic neuroendocrine tumors (NETs) that overexpress the somatostatin receptor 2 (SSTR2) [1]. This therapy employs [^177^Lu]Lu-[DOTA-Tyr^3^]octreotate (^177^Lu-DOTA-TATE), in which somatostatin analogues are conjugated with radionuclides and administered systemically. Upon binding of ^177^Lu-DOTA-TATE to SSTR2 expressed on the tumor cell membrane, the complex is internalized and induces DNA damage following radio-active decay, of which double-strand breaks (DSBs) are most cytotoxic [2]. While PRRT improves survival and quality of life, complete cures remain rare and its therapeutic potential is restricted by dose-limiting toxicity [3, 4]. Thus, further refinement of this treatment is required to enhance its clinical outcome.

A widely used approach for improving PRRT efficacy involves combination treatment with radiosensitizing agents that potentiate radiation-induced cytotoxicity. These radiosensitizers typically function by either amplifying radiation-induced DNA damage or impairing DNA damage repair [5]. An example of a broader-spectrum radiosensitizer is the inhibition of heat shock protein 90 (HSP90), a molecular chaperone protein of the HSP family that plays a central role in protein folding, stability and homeostasis. HSP90 regulates the stability of over a hundred client proteins, including oncogenic signaling molecules and key DNA damage response (DDR) components, thereby promoting cellular survival and stress adaptation [6, 7]. Moreover, HSP90 is frequently overexpressed in various cancers, including NETs, highlighting its potential as a target for radiosensitization to PRRT [8, 9].

Previous studies have demonstrated that HSP90 inhibitors enhance the efficacy of external beam radiotherapy (EBRT), primarily through inhibition of radiation-induced survival pathways such as the phosphatidylinositol 3-kinase (PI3K)/protein kinase B(Akt)- and microtubule associated protein kinase (MAPK)/extracellular signal-regulated kinase(ERK)-signaling pathways, as well as suppression of the DDR [10]. While HSP90 inhibition also has been shown to potentiate PRRT, the precise mechanisms underlying its sensitizing effects remain unclear [11–13]. Given the fundamental differences in radiation kinetics between EBRT and PRRT, including differential dose rates and exposure timings, the mechanism of radiosensitization by HSP90 inhibition possibly varies between these modalities.

To better understand how these treatments synergize and use this information to potentially increase their efficacy, we investigated the mechanistic basis of HSP90 inhibition as a radiosensitizing strategy for PRRT.

## Materials & methods

### Cell culture

Human small intestine NET cell-line GOT1 was kindly gifted by Ola Nilsson (Sahlgrenska Cancer Center, University of Gothenburg, Sweden) [14] and cultured in RPMI 1640 (Gibco) supplemented with 10% fetal calf serum (FCS), 1% penicillin-streptomycin (PS), 5 mM L-glutamine (Stemcell Technologies), 5 µg/mL human insulin (Sigma Aldrich) and 5 µg/mL human transferrin (Sigma Aldrich, T8158). Human pancreatic NET cells BON1-SSTR2 were generated as described previously [15] and maintained in DMEM/F12 (Gibco), supplemented with 10% FCS and 1% PS in presence of 500 µg/mL G418 (Invitrogen). Cells were maintained at 37 °C in a humidified incubator with 5% CO2.

### EBRT, PRRT and ganetespib

EBRT was applied by X-ray irradiation with a RS320 cabinet irradiator (X-strahl) at a dose rate of 1.6 Gy/min. Lu-Mark (IDB Holland) was used for in-house production of ^177^Lu-DOTA-TATE, which met standard characteristics for patient treatment (specific activity 53 MBq/nmol, radiometal incorporation > 95% and radiochemical purity > 90%). Ganetespib (STA-9090; Selleckchem, S1159) was stored as a 10 mM stock solution in DMSO at −80 °C until further use.

### Western blotting

GOT1 and BON1-SSTR2 cells were treated with ganetespib (10 or 25 nM) or vehicle and harvested in Laemmli buffer (4% SDS, 20% glycerol, 120 mM Tris pH 6.8) at 0, 0.5, 2, 6, 24 and 72 h post-treatment. Protein concentrations were determined by Lowry method. A total of 10 µg (GOT1) or 5 µg (BON1-SSTR2) protein was loaded per lane on precast Mini-PROTEAN TGX 4–15% gels (BioRad, 4561084) and gel electrophoresis was run at 100 V. Proteins were blotted onto a PVDF Transfer Membrane (Merck Millipore, IPVH00010) for 1 h at 100 V in blotting buffer (1X Tris Glycine buffer with 20% methanol). Membranes were blocked in PBS + 0.05% Tween (Sigma-Aldrich) + 3% skimmed milk (Merck Millipore) and incubated with primary antibodies rat anti-HSP70 (Cell Signaling Technology, 4873 S, 1:1000) and mouse anti-actin (Millipore, LV1547855, 1:500.000) at 4 °C overnight. After washing, membranes were incubated for 1 h with secondary antibodies horseradish peroxidase-conjugated polyclonal rabbit-anti-rat (Dako, P0459, 1:2000) and horseradish peroxidase-conjugated AffiniPure^™^ sheep-anti-mouse IgG (Jackson ImmunoResearch, 515-035-003, 1:2000) in blocking buffer at RT. Protein bands were detected by enhanced chemiluminescence (ECL) substrate and imaged on an Amersham Imager 600 (GE Healthcare Life Sciences). The experiment was performed as 3 independent biological replicates.

### Viability assay

Cells were incubated with ^177^Lu-DOTA-TATE (1 MBq/mL, 1.9 × 10^−8^M) or vehicle in suspension in culture medium (2 × 10^5^ cells/mL) in 15 mL tubes. After 4 h, radioactive medium was washed away and cells were seeded into white walled, clear flat bottom 96-well plates at a density of 3 × 10^4^ (GOT1) or 2 × 10^3^ (BON1-SSTR2) cells/well in 200 µL, in presence of ganetespib (1, 5, 10, 25 or 50 nM) or vehicle. In parallel, cells were first seeded into 96-well plates, pre-treated with ganetespib for 2 h and subsequently irradiated by EBRT (2 Gy). For BON1-SSTR2 cells, ganetespib was removed after 24 h and replaced with fresh medium. After 7 days, metabolic activity was measured by CellTiter-Glo^®^ 2.0 Assay (Promega, G9241). In short, 100 µL of CellTiter-Glo^®^ 2.0 Reagent was added per well and plates were incubated for 10 min at RT. Luminescence was measured on the Spectramax iD3 microplate reader (Molecular Devices). Experiments were performed as 3 independent biological replicates each with technical triplicates.

### Cell death assay

Cells were incubated with ^177^Lu-DOTA-TATE (1 MBq/mL, 1.9 × 10^−8^M) or vehicle in suspension in culture medium (2 × 10^5^ cells/mL). After 4 h, radioactive medium was washed away and cells were seeded into 6-well plates at a density of 1 × 10^6^ (GOT1) or 3.5 × 10^5^ (BON1-SSTR2) cells/well, in presence of ganetespib (10 or 25 nM) or vehicle. In parallel, cells were first seeded into 6-well plates, pre-treated with ganetespib for 2 h and subsequently irradiated by EBRT (2 Gy). For BON1-SSTR2 cells, medium was refreshed after 24 h to remove ganetespib. At day 3, live and dead cells were collected and incubated with SYTOX^™^ AADvanced^™^ Dead Cell Stain (1 µM) for 5 min at RT. Fluorescent signal was detected by flow cytometry on a LSRFortessa flow cytometer (BD Biosciences). Gating and analysis were performed using FlowJo^™^ software (BD Biosciences). Experiments were performed as 3 independent biological replicates.

### RAD51 immunofluorescent staining and foci quantification

GOT1 and BON1-SSTR2 were seeded on glass coverslips in 12-well plates and treated with vehicle, 10 or 25 nM ganetespib 2 h prior to irradiation (5 Gy). To label replicating cells, samples were incubated with 30 µM 5-ethynyl-2’-deoxyuridine (EdU) for 3 (GOT1) or 1 h (BON1-SSTR2) prior to fixation. At 2 and 6 h after EBRT, cells were pre-extracted on ice for 1 min in pre-extraction buffer (0.5% Triton X-100, 20 mM HEPES-KOH [pH7.9], 50 mM NaCl, 3 mM MgCl_2_, 300 mM sucrose), followed by fixation in 2% PFA for 15 min. Samples were blocked in PBS + 3% bovine serum albumin (BSA) and permeabilized in PBS + 0.1% Triton X-100. Then, cells were incubated for 30 min at RT with a reaction mix consisting of 40 mM Tris buffer, 4 mM CuSO_4_ (Sigma), 30 µM Atto 488 azide (ATTO-TEC) and 4 mM ascorbic acid (Sigma), protected from light. For immunostaining, cells were incubated at RT in PBS + 0.5% BSA + 20 mM glycine with primary RAD51 antibody (homemade rabbit polyclonal clone #2307, 1:10.000) for 1.5 h. Cells were again washed in PBS + 0.1% Triton X-100 and incubated with secondary Goat-anti-rabbit IgG Alexa Fluor 594 (Thermofisher, A-11012, 1:1000) in PBS + 0.5% BSA + 20 mM glycine for 1 h in the dark. Coverslips were mounted on microscope slides using Vectashield (Vector Laboratories) containing DAPI. Imaging of RAD51 foci was performed on a TCS SP5 confocal microscope (Leica) using a 63x oil immersion objective and excitation lasers at 405 nm (DAPI), 488 (EdU) and 561 (RAD51). Images were acquired as Z-stacks from 4 different fields of view per condition.

Image processing and quantification of RAD51 foci per EdU-positive nucleus were conducted using homemade scripts in FIJI [16]. In short, EdU-positive cells were segmented by thresholding the 488 nm channel to define the region of interest. Within each region of interest, RAD51 foci were segmented using the 561 nm channel, with predefined minimum and maximum foci sizes and intensities. At least 25 cells were analyzed per condition per replicate, and experiments were performed as 3 independent biological replicates.

### 53BP1 immunofluorescent staining and foci quantification

GOT1 and BON1-SSTR2 cells were plated in 12-well plates on glass coverslips. For EBRT, cells were incubated with vehicle, 10 or 25 nM ganetespib 2 h prior to irradiation (2 Gy). For PRRT, adherent cells were incubated for 4 h with ^177^Lu-DOTA-TATE (1 MBq/mL, 1.9 × 10^−8^M), after which the radioactive medium was replaced with medium containing ganetespib (10 or 25 nM) or vehicle. At 30 min, 6 and 24 h after radiation treatment, cells were fixed in 2% paraformaldehyde (PFA) for 15 min. For immunostaining, cells were permeabilized in PBS + 0.1% Triton X-100. Samples were blocked in PBS + 0.5% BSA + 20 mM glycine and incubated with primary antibody for 53BP1 (Novus Biologicals, NB100-904, 1:1000) in blocking buffer for 1 h at RT. Cells were then washed with PBS + 0.1% Triton X-100 and incubated in secondary Goat-anti-rabbit IgG Alexa Fluor 594 (Thermofisher, A-11012, 1:1000) in blocking buffer for 1 h at RT in the dark. Samples were mounted using Vectahield (Vector Laboratories) containing DAPI. Imaging of 53BP1 foci was done on a TCS SP5 confocal microscope (Leica) using a 63x oil immersion objective and 405 nm (DAPI) and 561 nm (53BP1) lasers.

Imaging and analysis were done in a similar manner to the RAD51 foci quantification. For analysis, nuclei were segmented in the DAPI channel, after which in each nuclear mask foci were segmented in the 53BP1 channel by thresholding, using a defined minimum and maximum foci size and intensity. At least 40 cells were analyzed per condition per replicate and experiments were performed as 3 independent biological replicates.

### End-joining assay

GOT1 or BON1-SSTR2 cells were plated in 6-well plates. As a positive control, 1 µM DNA-PKcs inhibitor AZD7648 (Medchemexpress) was added at the moment of seeding. After 24 h, treatment with 10 or 25 nM ganetespib was started. Assessment of the relative amount of classical non-homologous end joining (c-NHEJ) and microhomology-mediated end joining (MMEJ) used by cells was done as described before [17]. In short, 2 h after starting ganetespib treatment, a linear DNA substrate (pDVG94 plasmid) was transfected into cells. Extrachromosomal DNA was isolated 2 days after transfection. Plasmid DNA was PCR-amplified and digested by using the restriction enzyme BstXI, DNA fragments were separated and visualized using gel electrophoresis in a 6% polyacrylamide gel in Tris/borate/ethylenediaminetetraacetic acid (TBE) buffer using ethidium bromide. The experiment was performed as 2 independent biological replicates.

### RNA sequencing

GOT1 cells were incubated in suspension ^177^Lu-DOTA-TATE (1 MBq/mL, 1.9 × 10^−8^M) or vehicle for 4 h and seeded in 6-well plates in presence or absence of 10 nM ganetespib. Total RNA was isolated at 24 and 72 h post-PRRT using the RNeasy Mini Kit (QIAGEN) according to the manufacturer’s protocol and stored at −80 °C before further processing. RNA concentration, purity and quality were assessed on a Nanodrop 2000 Spectrophotometer (Thermo Scientific) and Bioanalyzer 2100 (Agilent). Library preparation and RNA sequencing were performed at Genomescan B.V. (Leiden, The Netherlands). In short, libraries were prepared using the NEBNext Ultra II Directional RNA Library Prep Kit (New England Biolabs). Clustering and RNA sequencing on the NovaSeq6000 (Illumina) was performed at an average sequencing depth of 28 million reads and 150 bp read length. Samples sent for RNA sequencing comprised 3 biological replicates per condition.

### RNA-seq processing, differential gene expression, and gene set enrichment analysis

The raw fastq files were pre-processed with fastp v0.23.2 [18]. Samples were then aligned using STAR v2.7.10a [19] against the human reference genome hs38 with ENSEMBL v107 annotation. Quality control was assessed using fastqc v0.11.9 (https://github.com/s-andrews/FastQC/) and RSeQC v5.0.1 [20, 21]. MultiQC v1.15.dev0 [22] was used to summarize the quality control of these two programs and the different steps of the data processing. Read counts overlapping the entire gene body were generated using htseq-count v2.0.2 [23].

The resulting gene-count matrices were analyzed using R (version 4.4). One sample (001, Control 24 h) showed a diverging expression profile and it was excluded from subsequent analyses. Differential gene expression between groups and conditions was analyzed using DESeq2 v1.44.0 [24] by two different models, with an adjusted p-value threshold of 0.05 and |Fold Change| ≥ 1.2. The first model used was “design = ~ group”, where group represents the combination of treatment and time point. In addition, an interaction model (“design = ~ radiation*inhibitor”) was used (only for samples at 24 h) to decouple the effects of PRRT and ganetespib monotherapies and evaluate the synergistic effects of both therapies in the combination treatment samples.

Gene Set Enrichment Analysis (GSEA) was performed using the R package fGSEA v1.30.0 [25] and MSigDB (v2023.2) human GO: Biological Process and GO: Molecular Function were used as a reference [26, 27]. Each comparison between groups and conditions was analyzed separately using the log2 Fold Change of all detected genes. Genes not present in the data were excluded from the background.

### Statistical analysis

Statistical analyses for all experiments, except for RNA sequencing, were performed in Graphpad Prism (version 9.4.0). Results are presented as mean ± standard error of the mean (SEM). For viability and cell death assays, statistical significance between treatments was determined by an unpaired t-test. For foci analysis experiments (53BP1, RAD51), a one-way ANOVA followed by Tukey’s multiple comparisons test was performed. P-values were considered significant if *p* < 0.05.

## Results

### Ganetespib activates the heat shock response and sensitizes NET cells to EBRT and PRRT

**Fig. 1:**
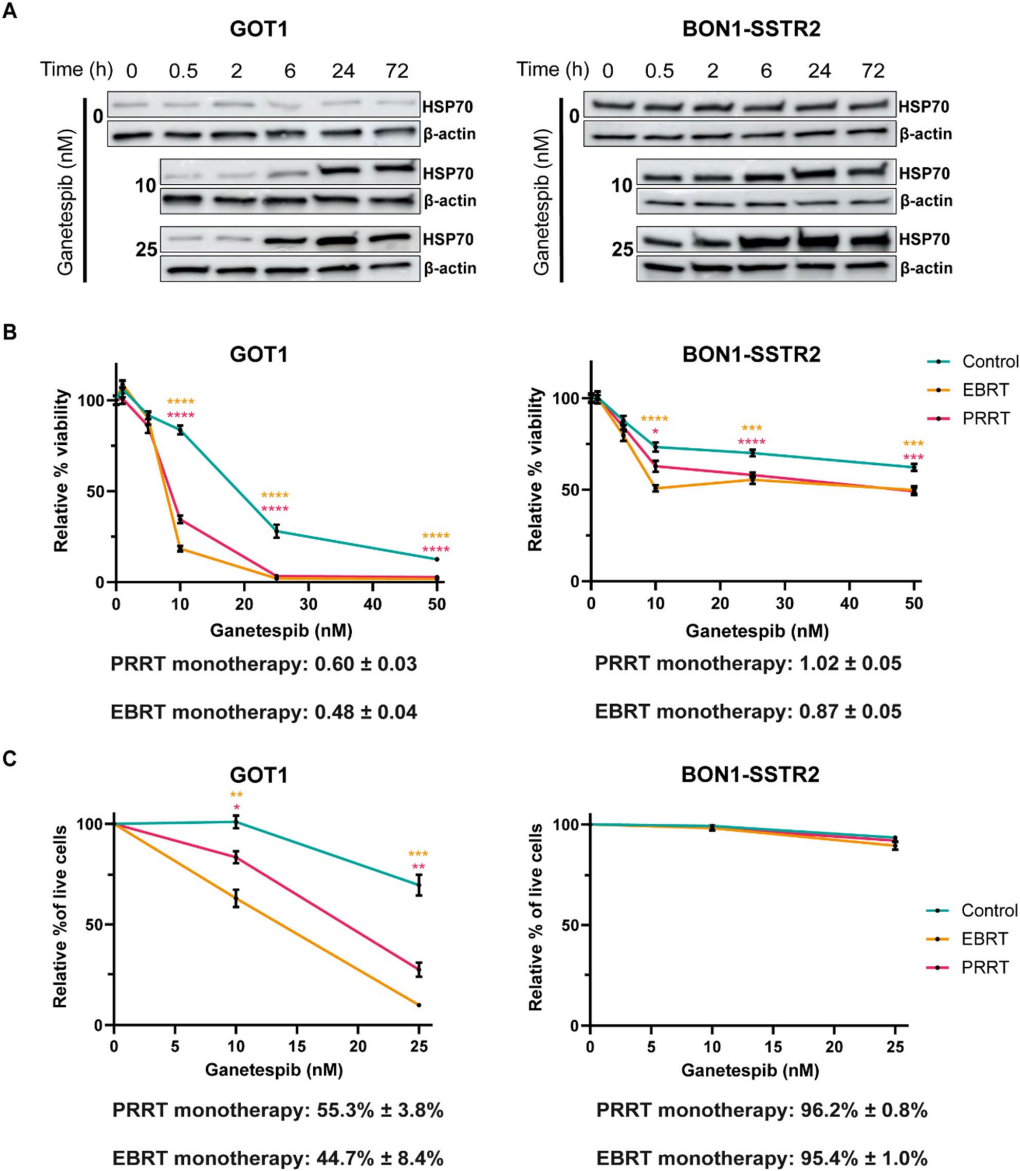
Efficacy of combination treatment of ganetespib with EBRT and PRRT.** (A)** Time-dependent induction of HSP70 protein expression after start of ganetespib treatment (10 or 25 nM) for GOT1 (left) and BON1-SSTR2 (right) cells. β-actin was used as loading control. **(B)** Viability assay for GOT1 (left) and BON1-SSTR2 (right) cells of a concentration range of ganetespib as monotherapy, or combined with 2 Gy EBRT or 1 MBq/mL PRRT 7 days post treatment initiation. Values are expressed as the percentage viability and normalized to their respective viability without ganetespib (0 nM). Data points represent the mean of 3 independent biological replicates with technical triplicates. Below the graphs, the viability of PRRT or EBRT monotherapy is depicted. **(C)** Assessment of cell death for GOT1 (left) and BON1-SSTR2 (right) cells after ganetespib (0, 10 or 25 nM) as monotherapy, or combined with 2 Gy EBRT or 1 MBq/mL PRRT 3 days post treatment initiation. Values are expressed as the percentage of living cells and normalized to their respective control condition (without ganetespib, 0 nM) for each curve. Data points represent the mean of 3 independent biological replicates. Below the graphs, the relative percentage of live cells of PRRT or EBRT monotherapy is depicted. All error bars represent the SEM. Asterisks indicate the level of significance of the difference for each corresponding point with control. *p ≤ 0.05, ** *p* ≤ 0.01, *** *p* ≤ 0.001, **** *p* ≤ 0.0001

To sensitize NET cell lines GOT1 and BON1-SSTR2 to ionizing radiation, we treated the cells with small molecule inhibitor ganetespib, which competitively binds to the N-terminal ATP-binding pocket of HSP90 [28]. HSP90 expression has been previously demonstrated in both GOT1 and BON1 cell lines [29]. To confirm effective HSP90 inhibition and assess its temporal dynamics, we characterized the cellular response to ganetespib according to HSP70 protein levels, which is an established biomarker for detection of HSP90 inhibition and the subsequent activation of the heat shock response (HSR) [30]. In both GOT1 and BON1-SSTR2 cells, HSP70 levels were increased following ganetespib treatment within 6 h, and remained elevated for 72 h (Fig. 1A), suggesting successful inhibition of HSP90 for (at least) 72 h.

To evaluate the radiosensitizing potential of ganetespib in our setup, we indirectly measured cell viability by evaluating metabolic activity seven days after starting the treatment. In GOT1 cells, PRRT and EBRT monotherapy resulted in a relative cell viability of 0.60 and 0.48 compared to control, respectively. In contrast, BON1-SSTR2 cells exhibited markedly lower sensitivity to both radiation treatments, with relative viabilities of 1.02 for PRRT and 0.87 for EBRT (Fig. 1B). In GOT1 cells, combination treatment with ganetespib and either PRRT or EBRT significantly reduced cell viability compared to ganetespib monotherapy (Fig. 1B). However, in BON1-SSTR2 cells, continuous ganetespib treatment was excessively toxic by itself (Supplementary Fig. 1 A). Therefore, we modified the treatment protocol by washing out ganetespib after 24 h of incubation and replacing it with drug-free medium. This resulted in a small but significant reduction in cell viability after seven days of combination treatment compared to ganetespib monotherapy treated cells (Fig. 1B).

To further validate whether the observed decrease in cell viability was attributable to cell death induction, we quantified cell death three days after treatment initiation by flow cytometry (Supplementary Fig. 1B). In GOT1 cells, a dose-dependent decrease in living cells was observed, which was significantly stronger in EBRT and PRRT treated cells compared to the ganetespib monotherapy (Fig. 1C). In contrast, in BON1-SSTR2 cells no such effect was observed. Additionally, consistent with the cell viability assay, these cells showed negligible sensitivity to ionizing irradiation itself (Fig. 1C).

### Treatment with Ganetespib has limited impact on the persistence of radiation-induced DSBs

**Fig. 2:**
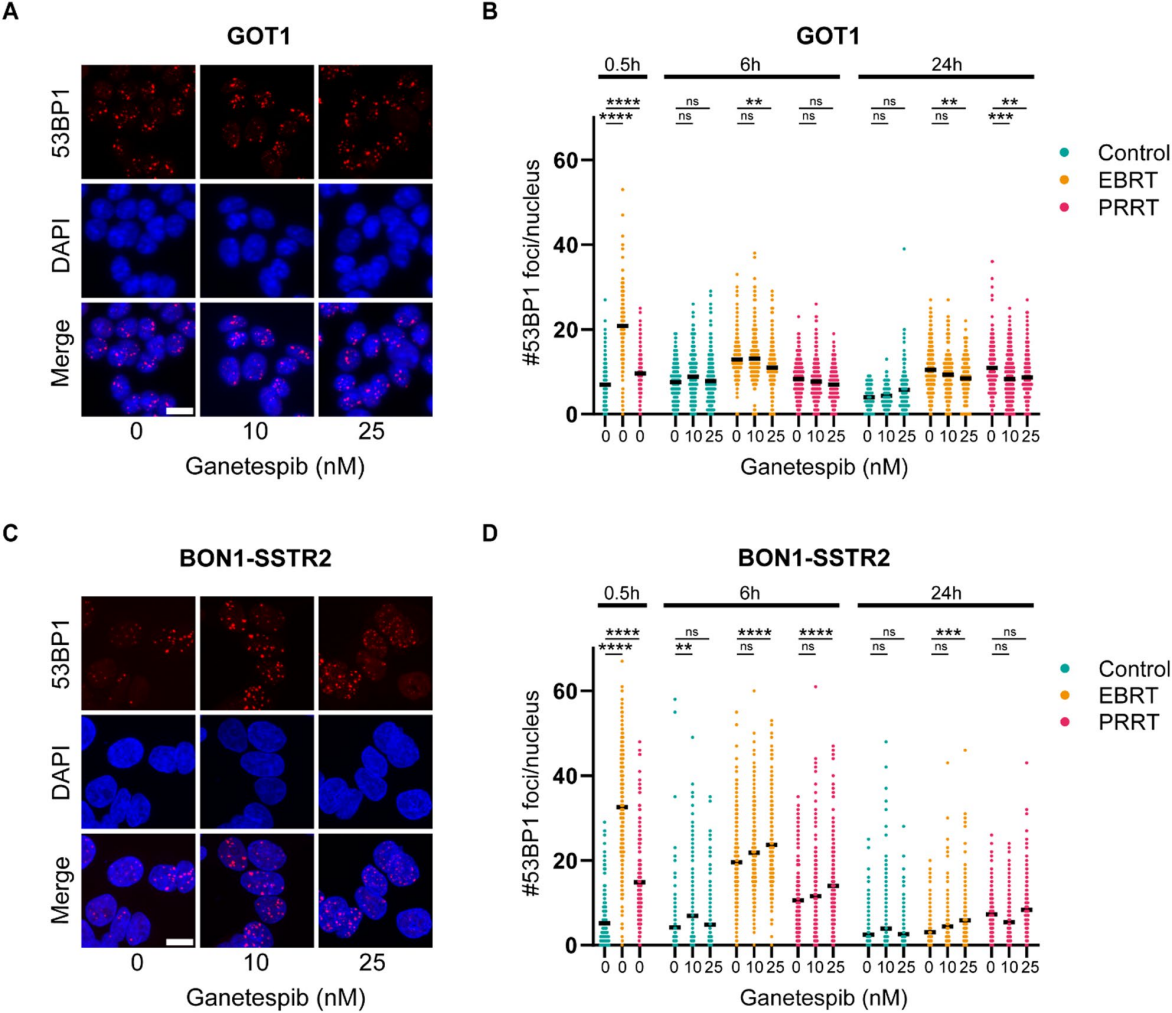
Effect of HSP90 inhibition on EBRT- and PRRT-induced 53BP1 foci. Cells were treated with 2 Gy EBRT or 1 MBq/mL PRRT and 10 or 25 nM ganetespib or vehicle. **A**,** C)** Representative images of 53BP1 foci (red) and cell nucleus (blue) 6 h post treatment initiation in GOT1 (**A**) and BON1-SSTR2 (**C**) cells. Scale bars indicate 10 μm. **B**,** D)** Quantification of 53BP1 foci per nucleus in GOT1 (**B**) and BON1-SSTR2 (**D**) cells at 0.5, 6 and 24 h after start of treatment. Figures represent data from 3 independent biological replicates pooled together. Black horizontal lines indicate the mean. Ns = not significant, ** *p* ≤ 0.01, *** *p* ≤ 0.001, **** *p* ≤ 0.0001

The radiosensitizing effect of HSP90 inhibition has previously been associated with inhibitory effects on the DDR, more specifically through inhibition of DSB repair [31–34]. To determine whether the observed increase in cell death following combined ganetespib and EBRT or PRRT was associated with elevated DNA damage as a result of perturbed DNA repair, we quantified p53 binding protein 1 (53BP1) foci as marker of DSB levels [35]. Following EBRT, both GOT1 and BON1-SSTR2 cells exhibited a significant increase in 53BP1 foci at 0.5 h post-irradiation, which gradually declined to baseline level within 24 h. In contrast, PRRT induced a milder but still significant increase in 53BP1 foci at 0.5 h post-incubation (Fig. 2B, D).

Ganetespib monotherapy did not increase the number of 53BP1 foci in GOT1 cells, nor did it enhance DSB levels when combined with EBRT or PRRT at the analyzed time-points (Fig. 2A, B). At the 24 h time-point, even a modest reduction in the number of 53BP1 foci was observed following ganetespib treatment in combination with EBRT and PRRT, though this difference was relatively small and therefore might lack biological relevance. In BON1-SSTR2 cells, a trend toward a dose-dependent increase in 53BP1 foci following ganetespib treatment was observed when combined with EBRT or PRRT; however, the differences were again relatively minor and not statistically significant across all conditions and time points (Fig. 2C, D). Together, these findings suggest that despite its radiosensitizing effect, ganetespib has a limited impact on overall DSB levels and persistence of DSBs after EBRT and PRRT under the tested conditions.

### HSP90 Inhibition impairs homologous recombination through decreasing irradiation-induced RAD51 foci formation

Since the effect of ganetespib on DSB persistence was not clearly visible, we further investigated the role of specific DSB repair pathways in the sensitization. Therefore, we analyzed the effect of ganetespib on homologous recombination (HR) and classical non-homologous end-joining (c-NHEJ), the two major DSB pathways.

**Fig. 3:**
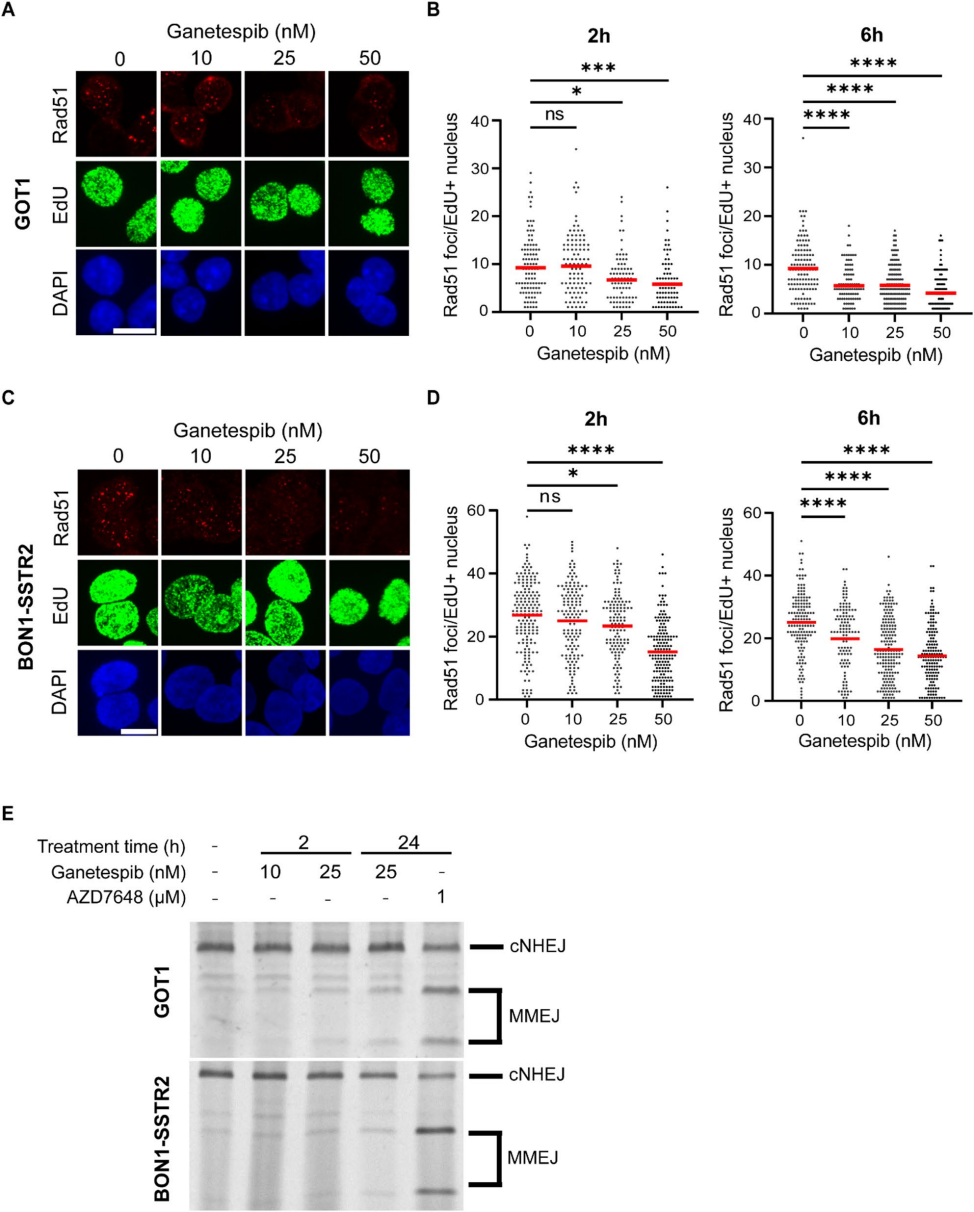
Functional analysis of the effect of ganetespib on DSB repair pathways HR and c-NHEJ.** A-D)** Assessment of HR in GOT1 and BON1-SSTR2 cells after EBRT (5 Gy) and ganetespib (0, 10, 25 or 50 nM). **A**,** C)** Representative images of RAD51 foci (red), EdU + cells (green) and cell nucleus (blue) 2 h post-irradiation in GOT1 (**A**) and BON1-SSTR2 (**B**) cells. Scale bars indicate 10 μm. **B**,** D)** Quantification of the number of RAD51 foci per EdU + nucleus in GOT1 (**B**) and BON1-SSTR2 (**D**) cells 2 and 6 h post-irradiation. Figures represent data from 3 independent biological replicates pooled together. Red horizontal lines indicate the mean and error bars indicate SEM. Ns = not significant, * *p* ≤ 0.05, *** *p* ≤ 0.001, **** *p* ≤ 0.0001. **E)** Assessment of the balance between c-NHEJ and MMEJ by end-joining assay, non-treated, after 2 h of ganetespib treatment (10 or 25 nM), or after 24 h of ganetespib (25 nM) or AZD648 (1 µM) treatment in GOT1 and BON1-SSTR2 cells

To determine the effect on HR, we analyzed the effect of ganetespib on RAD51 foci formation after irradiation, specifically in S-phase cells by selecting for EdU-positive nuclei (Fig. 3A, C). RAD51 specifically accumulates on DSBs being repaired by HR. Since the previously used radiation conditions (2 Gy EBRT; 1 MBq/mL PRRT) did not induce a detectable number of RAD51 foci (data not shown), we focused on a higher dose of EBRT (5 Gy) as a general indicator for HR functioning. Irradiated GOT1 and BON1-SSTR2 cells showed a clear presence of RAD51 foci both at 2 h and 6 h post-irradiation in proliferating cells. Treatment with ganetespib resulted in a significant and dose-dependent decrease in the number of RAD51 foci, with the most pronounced effect observed at 6 h (Fig. 3B, D), indicating that HR is (partially) blocked by ganetespib.

Subsequently, effects on DSB repair by c-NHEJ were assessed by an end-joining assay. This assay measures the balance between c-NHEJ and microhomology-mediated end joining (MMEJ) using an exogenous repair product. Positive control, DNA-PKcs inhibitor AZD7648 induced a profound shift from c-NHEJ to MMEJ products, indicative of a strong inhibitory effect on c-NHEJ. However, 2 h treatment with ganetespib pre-transfection did not induce a visible shift in repair products for both cell lines (Fig. 3E). Increasing the duration of ganetespib treatment before transfection to 24 h as a control for treatment scheme-dependent effects, also did not induce a shift to MMEJ. These results, together with the 53BP1 foci quantification, indicate that ganetespib impairs HR in GOT1 and BON1-SSTR2 cells, but this has a limited effect on the overall repair of DSBs induced by EBRT and PRRT.

### Transcriptomic analysis shows activation of the heat shock response upon HSP90 Inhibition

Given the significant effect on overall survival, but limited impact of HSP90 inhibition on DSB repair following both EBRT and PRRT, we hypothesized that additional cell mechanisms may contribute to the observed radiosensitizing effects. To gain deeper insight into the cellular response to HSP90 inhibition in the context of PRRT, we performed RNA sequencing in GOT1 cells, which showed the strongest degree in radiosensitization (Fig. 1B). The effect of ganetespib, PRRT and combination treatment was analyzed after 24 and 72 h of treatment by differential gene expression analysis.

**Fig. 4:**
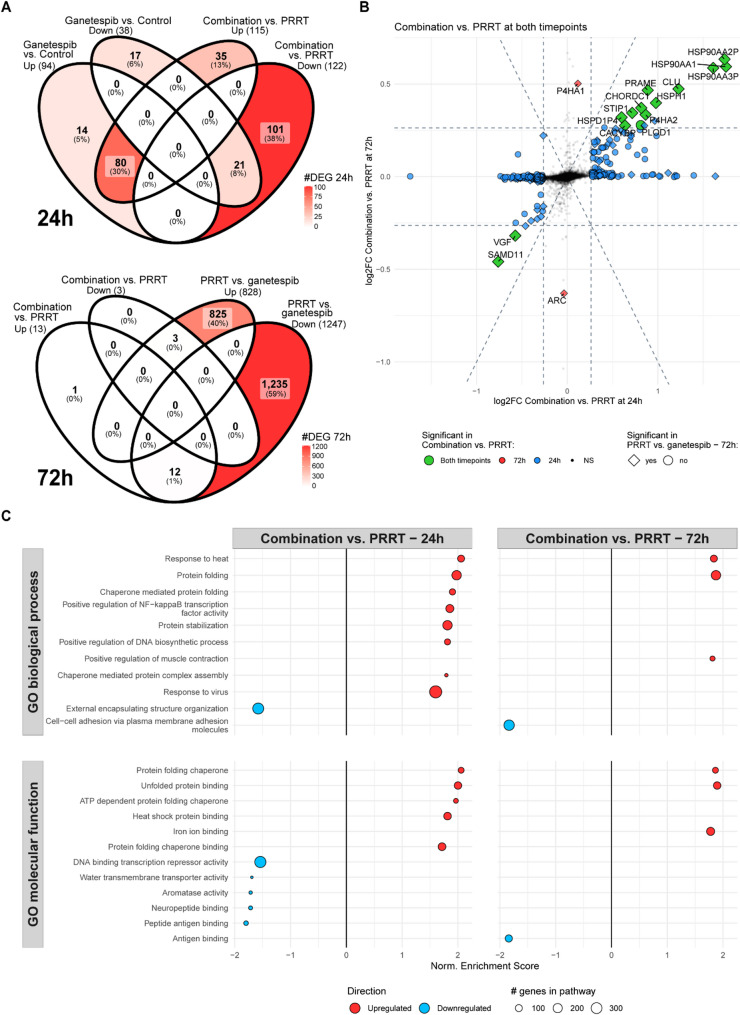
Transcriptomic analysis of the response of GOT1 cells to PRRT and ganetespib at 24 and 72 h.** (A)** Venn diagrams showing the number and overlap of DEGs after ganetespib monotherapy (ganetespib vs. control) and in the context of PRRT (combination vs. PRRT) after 24 h of treatment, and the effect of HSP90 inhibition in the context of PRRT (combination vs. PRRT) relative to both monotherapies (ganetespib vs. PRRT) after 72 h of treatment. **(B)** Scatter plot showing non-significant (black) and significant DEGs between combination and PRRT at 24 h (blue) and 72 h (red) or both time points (green). Genes that were also differentially expressed between both monotherapies (ganetespib vs. PRRT) at 72 h are indicated with a diamond. The horizontal and vertical dashed lines denote the minimum log2FoldChange threshold set for significance (log2(1.2)). The diagonal dashed lines denote a slope of 1. **(C)** GSEA results for the comparison of combination and PRRT using GO database at 24 and 72 h after treatment. Circle size indicates the number of genes represented in the pathway. Circle color indicates upregulation (red) or downregulation (blue) of the pathway

To evaluate the transcriptional effect of ganetespib monotherapy, differentially expressed genes (DEGs) were identified by comparing ganetespib treatment to control. Similarly, the effect of ganetespib in the context of PRRT was assessed by comparing combination treatment to PRRT monotherapy. After 24 h of treatment, there was an overlap of 80 (62%) upregulated and 21 (15%) downregulated DEGs being shared between the two comparisons (Fig. 4A). To focus on DEGs specific to ganetespib treatment in presence of PRRT, non-overlapping DEGs were further analyzed by an interaction model, that uses the effects of PRRT and ganetespib monotherapies as covariates. The interaction effect revealed no significant DEGs, suggesting that at 24 h, the expression changes observed in the combination treatment were the additive effects of PRRT and HSP90 inhibition, with no synergistic effects observed (Supplementary Fig. 2 A).

Comparing the effects of ganetespib in the context of PRRT at both 24 and 72 h time points revealed a similar response that became much more attenuated after 72 h, as indicated by the number of significant DEGs identified (237 DEGs at 24 h vs. 16 DEGs at 72 h) and the generally lower fold change observed at 72 h (Fig. 4B). Due to the lack of a control sample at 72 h, interaction model analysis could not be performed at this time point. Instead, the effect of HSP90 inhibition in the context of PRRT (combination vs. PRRT) relative to both monotherapies (ganetespib vs. PRRT) was analyzed for overlapping DEGs. This comparison showed a high level of concordance, with 15 out of 16 (94%) genes being shared between conditions (Fig. 4A). For all these comparisons the top significant DEGs were mainly upregulated canonical chaperone genes involved in the compensatory HSR upon HSP90 inhibition (e.g. *HSP90AA2P*,* HSP90AA3p*,* HSP90AA1*), again validating the biological activity of ganetespib at the analyzed time points.

GSEA of DEGs between combination and PRRT monotherapy revealed exclusive enrichment of pathways related to HSP90 function, including response to heat and protein folding, which was the strongest at 24 h (Fig. 4C). At this time point, similar affected pathways were found after ganetespib mono-treatment (Supplementary Fig. 2B). Taken together, these results demonstrate that HSP90 inhibition induces a detectable transcriptomic response at 24 h which diminishes by 72 h, likely reflecting its predominant mode of action at the protein level. The transcriptomic profile of the combination treatment suggests an orthogonal and functionally distinct transcriptomic response. This likely results in an additive, rather than synergistic, effect of PRRT and HSP90 inhibition on cell survival.

## Discussion

HSP90 inhibition has been established as an effective radiosensitization strategy in the context of EBRT across various cancer types [10]. More recently, HSP90 inhibition has shown promising results in enhancing sensitivity of NETs to PRRT employing ^177^Lu-DOTA-TATE, although the underlying mechanisms remain incompletely understood [11–13]. In this study, we aimed to elucidate the pathways contributing to the radiosensitizing effect of HSP90 inhibition to PRRT.

Consistent with previous studies, we showed that the HSP90 inhibitor ganetespib enhances the efficacy of both EBRT and PRRT in NET cell lines GOT1 and BON1-SSTR2. Notably, the degree of radiosensitization varied between the two models, with BON1-SSTR2 cells exhibiting pronounced toxicity to ganetespib monotherapy, likely reflecting a stronger dependency on HSP90 function. To mitigate this toxicity, we modified the treatment regimen by removing the inhibitor after 24 h, which indeed reduced monotherapy toxicity and resulted in a small but significant radiosensitizing effect. Further optimization of ganetespib treatment strategies, e.g. by adjusting exposure duration or using a lower concentration range of the inhibitor, may help expand the therapeutic window of HSP90 inhibition.

Next, we validated whether the observed decrease in cell viability was attributable to enhanced radiation-induced cell death. In GOT1 cells, a dose-dependent increase in cell death was detected, whereas no such effect was observed in BON1-SSTR2 cells. This aligns with the differences in radiosensitization between cell lines as observed in the viability assays, and may also reflect variations in the timing of cell death induction between the two cell lines. Indeed, BON1-SSTR2 cells proliferate rapidly (doubling time ~ 34 h [36]), while GOT1 cells much more slowly (>18 days [14]). As consequence, it is possible that by 72 h post-treatment initiation, the surviving fraction BON1-SSTR2 cell population may have undergone sufficient proliferation to mask the effects of initial cell death, whereas in the slower-proliferating GOT1 cells, these effects remain detectable. Alternatively, the reduced viability in BON1-SSTR2 cells following combination treatment may primarily reflect decreased proliferation or metabolic activity rather than increased radiation-induced cell death. Finally, differences in radiosensitization could correlate with intrinsic radiosensitivity of cell lines, as both EBRT and PRRT monotherapy were less toxic for BON1-SSTR2 cells than for GOT1 cells.

Since DSBs are the primary determinant of radiation-induced effects, targeting DDR components, likely involved in radiosensitization mechanisms, is a widely studied approach to enhance radiation sensitivity [37]. The two primary DSB repair pathways are HR and c-NHEJ. HR is a high-fidelity repair mechanism that utilizes the homologous sister chromatid as a template for repair and is therefore restricted to cells in the S and G2 phases of the cell cycle. In contrast, c-NHEJ mediates repair by directly ligating the broken DNA ends without the need of a homologous template, making it a more error-prone but widely utilized mechanism throughout all cell cycle phases [38]. Both disruption of c-NHEJ and HR have demonstrated radiosensitizing potential [15, 39, 40]. As HSP90 stabilizes proteins at various stages of DNA damage detection, repair and cell cycle check points, it is likely that HSP90 inhibition affects the DDR in a multi-target manner. Previous studies have shown that HSP90 inhibition leads to reduction of the overall cellular levels of key HR proteins such as BRCA2 and RAD51 [32, 33, 41]. However, since these proteins are predominantly expressed during S and G2 phases of the cell cycle, their downregulation at the population level may largely reflect reduced proliferation and a consequent decrease in the proportion of HR-competent cells, rather than a direct impairment of HR itself. To address this, we specifically assessed the effect of ganetespib on HR in EdU-positive (S/G2 phase) nuclei and confirmed that HSP90 inhibition indirectly impairs HR as indicated by a reduced number of RAD51 foci. In contrast, limited data is available showing effects of HSP90 inhibition on c-NHEJ, or more indirectly by showing a decrease in levels of c-NHEJ mediators such as DNA-PKcs [41–43]. Our end-joining assay did not show a shift from c-NHEJ to MMEJ as an alternative pathway after treatment, suggesting that c-NHEJ is not affected by treatment with radiosensitizing doses of ganetespib.

Despite HR inhibition, ganetespib had little impact on overall DSB persistence following EBRT and PRRT as reflected by the minimal changes in 53BP1 foci numbers. One possible explanation is that the inhibitory effect was insufficient to substantially impair overall DSB repair. Alternatively, HR may contribute too little to DSB repair under the examined conditions to produce a detectable effect, which partly aligns with the established predominance of c-NHEJ as the primary DSB repair pathway in mammalian cells [44]. This may account for the modest, dose-dependent increase in 53BP1 foci observed in BON1-SSTR2 cells, as their higher proliferation rate likely makes them more reliant on HR compared to the slower dividing GOT1 cells. While our analysis confirmed that ganetespib impairs HR following EBRT, it remained unclear whether similar mechanisms contribute to radiosensitization in the context of PRRT, which differs in radiation delivery and kinetics. To address this, we performed RNA sequencing in GOT1 cells, which we selected for their pronounced response to PRRT combined with HSP90 inhibition. To specifically isolate the impact of HSP90 inhibition on cellular pathways during PRRT, our analysis focused primarily on transcriptomic differences between PRRT monotherapy and the PRRT-HSP90 inhibitor combination.

It is well established that HSP90 inhibition activates a compensatory HSR, which is transcriptionally regulated by heat shock factor 1 (HSF1). In response to HPS90 inhibition, HSF1 upregulates a set of HSR-related genes and induces molecular chaperones such as HSP70, to restore proteostasis [45]. Our transcriptomic analysis indeed confirmed a robust induction of this compensatory HSR after 24 h of treatment. However, by 72 h, the transcriptional response was markedly reduced, with only 16 DEGs remaining between PRRT and combination treatment. Moreover, the overall transcriptomic response to the combination treatment was relatively modest, characterized by low-magnitude fold changes across most genes. This limited transcriptional signature is likely a reflection of HSP90’s primary mode of action at the post-translational level. Considering the temporal difference between transcriptomic and proteomic responses, comprehensive proteomic analysis following HSP90 inhibition may offer deeper insight into the dynamic regulation of affected pathways.

GSEA between PRRT and combination treatment solely showed enrichment of pathways associated with HSP90 function, such as protein folding and response to heat. As GSEA between control and HSP90 monotherapy showed enrichment of similar pathways, it is likely that the increased efficacy after combination treatment is an additive result of HSP90 inhibition and PRRT. These findings suggest that the radiosensitizing effect of HSP90 inhibition is not attributed to synergistic disruption of specific signaling pathways, but instead is caused by a pleiotropic effect resulting from loss of HSP90 function.

The pleiotropic nature of HSP90 inhibition is supported by its broad list of client proteins and central role in managing cellular stress, particularly in tumor cells [10]. For example, HSP90 is essential for the stability and function of proteins regulating cell cycle progression and checkpoint control, such as cyclin-dependent kinases CDK4 and CDK1 [34], and upstream Serine/threonine-protein kinase (ATR) [46]. Degradation of these proteins upon HSP90 inhibition results in cell cycle arrest at the G2/M phase, the stage at which cells are most sensitive to ionizing irradiation [47]. In addition, HSP90 supports multiple radioresistance pathways. In the PI3K/Akt pathway, it maintains key proteins like Akt [48–50], PDK1 and mTOR [50, 51], resulting in downregulation of this signaling axis upon inhibition of HSP90. Similarly, the MAPK/ERK pathway is disrupted upon HSP90 inhibition through depletion of Raf and ERK proteins [52, 53]. Importantly, in certain cancer types, inhibition of the PI3K/Akt pathway has been shown to induce compensatory activation of the MAPK/ERK pathway, thereby sustaining cell survival despite inhibition of PI3K [54]. This compensatory mechanism highlights the advantage of HSP90 inhibition as a multitarget approach, capable of synchronously impairing multiple survival and radioresistance pathways.

## Conclusions

Taken together, our data suggests that the radiosensitizing effect of HSP90 inhibition is unlikely to be mediated by the suppression of a single signaling pathway. Instead, it likely results from the simultaneous, moderate disruption of several interconnected pathways – including those regulating cell survival, proliferation, radioresistance, and cell cycle progression – ultimately leading to a synergistic reduction in tumor cell viability when combined with PRRT.

## Supplementary Information

Supplementary Material 1.

## Data Availability

Data is available upon reasonable request to the corresponding author. The RNA-sequencing dataset generated in the current study has been deposited in the NCBI Sequence Read Archive (SRA) under BioProject accession number PRJNA1321722 (https://eur01.safelinks.protection.outlook.com/?url=https%3 A%2 F%2Fwww.ncbi.nlm.nih.gov%2Fsra%2FPRJNA1321722&data=05%7C02%7Cp.engbers%40erasmusmc.nl%7C6f73a3eeb67c464e8c1308ddf0055fa2%7C526638ba6af34b0fa532a1a511f4ac80%7C0%7C0%7C638930628058766511%7CUnknown%7CTWFpbGZsb3d8eyJFbXB0eU1hcGkiOnRydWUsIlYiOiIwLjAuMDAwMCIsIlAiOiJXaW4zMiIsIkFOIjoiTWFpbCIsIldUIjoyfQ%3D%3D%7C0%7 C%7 C%7 C&sdata=SN%2 F%2FF9Mt9gf4i0aP7YP1FIYJZlxl0Z%2BOPqQbWb3bHiM%3D&reserved=0).

## Abbreviations

Akt: Protein kinase B
ATR: Serine/threonine-protein kinase
CDK: Cyclin-dependent kinase
c-NHEJ: Classical non-homologous end joining
DEG: Differentially expressed gene
DDR: DNA damage response
DSB: Double-stranded break
EBRT: External beam radiotherapy
EDU: 5-ethynyl-2’-deoxyuridine
ERK: Extracellular signal-regulated kinase
GSEA: Gene set enrichment analysis
HR: Homologous recombination
HSF1: Heat shock factor 1
HSP90: Heat shock protein 90
HSR: Heat shock response
MAPK: Microtubule associated protein kinase
MMEJ: Microhomology-mediated end joining
mTOR: Mammalian target of rapamycin
NETs: Neuroendocrine tumors
PDK1: 3-phosphoinositide-dependent protein kinase-1
PI3K: Phosphatidylinositol 3-kinase
PRRT: Peptide receptor radionuclide therapy
SSTR2: Somatostatin receptor 2
53BP1: p53 binding protein 1
^177^Lu-DOTA-TATE: [^177^Lu]Lu-[DOTA-Tyr^3^]octreotate
