# Supplementary material for: Radiosensitization of NET cells by HSP90 inhibitor ganetespib is mediated through pleiotropic stress responses
Source: EJNMMI Res. 2025 Nov 21;15:146. doi: 10.1186/s13550-025-01346-z (PMC12748489; doi:10.1186/s13550-025-01346-z)
Supplement: Supplementary file 1 — Supplementary Material 1. [file 13550_2025_1346_MOESM1_ESM.docx]

## Radiosensitization of NET cells by HSP90 inhibitor ganetespib is mediated through pleiotropic stress responses

**Pleun A.M. Engbers^a,b,*^, Thom G.A. Reuvers^a,b,*^, José María Heredia-Genestar^a,b^, Jiang Chang^a^, Nicole S. Verkaik^a^, Mariangela Sabatella^a,b^ and Julie Nonnekens^a,b^**

^a^ Department of Molecular Genetics, Erasmus MC Cancer Institute, Erasmus University Medical Center Rotterdam, The Netherlands; ^b^ Department of Radiology and Nuclear Medicine, Erasmus MC Cancer Institute, Erasmus University Medical Center Rotterdam, The Netherlands; ^*^ These authors contributed equally.

**Corresponding author:** Julie Nonnekens ([j.nonnekens@erasmusmc.nl](mailto:j.nonnekens@erasmusmc.nl))

**Address:** Erasmus University Medical Center, Dr. Molewaterplein 40, 3015GD, Rotterdam, The Netherlands

**Appendix A. Supplementary figures**


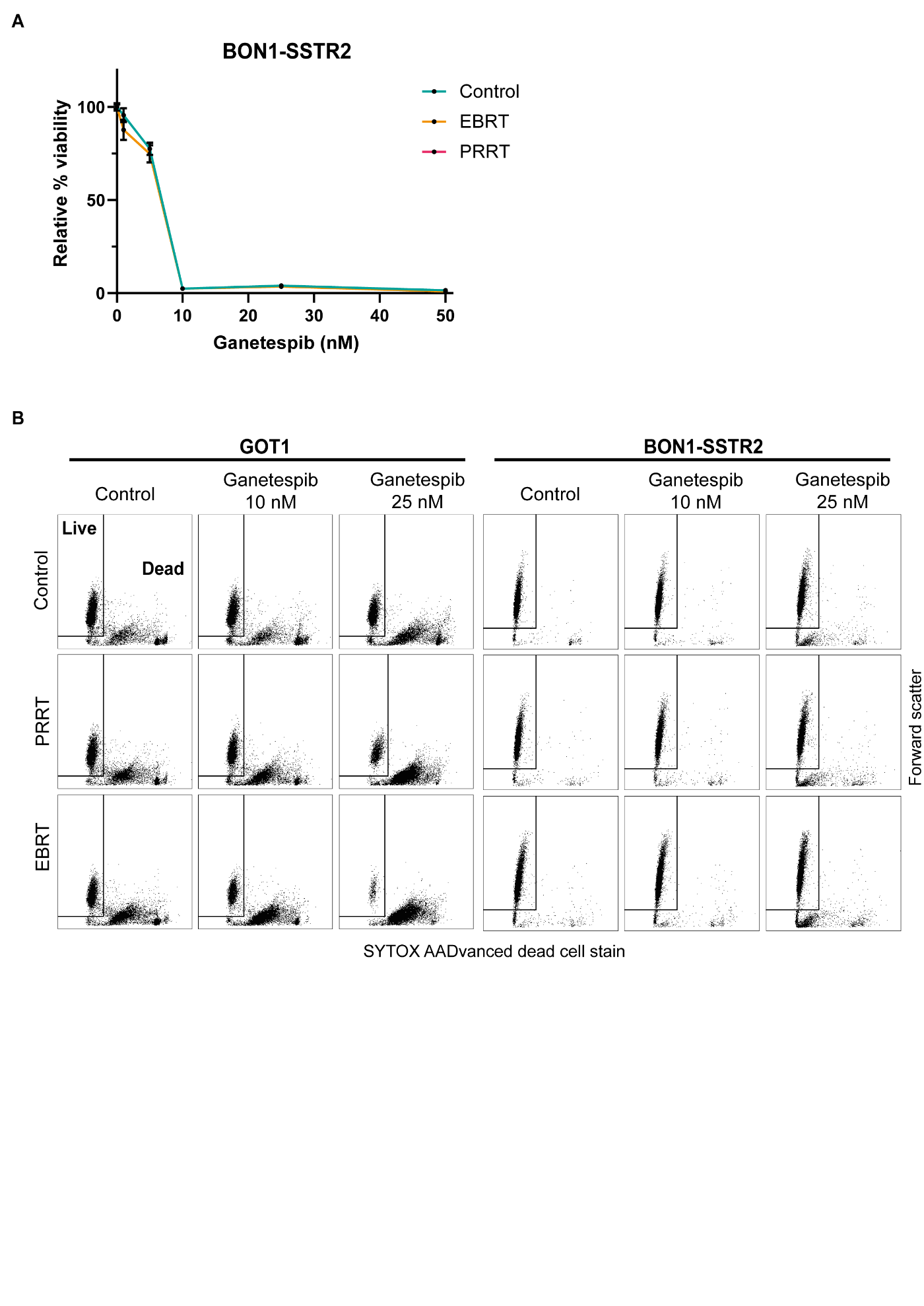


**Supplementary Figure 1. Efficacy of combination treatment of ganetespib with EBRT and PRRT. A)** Viability assay for BON1-SSTR2 cells of a concentration range of ganetespib as monotherapy or combined with 2 Gy EBRT or 1 MBq/mL PRRT 7 days post-treatment initiation, with continuous treatment of ganetespib. All curves are normalized to their respective viability without ganetespib (0 nM). Data points represent the mean of 3 independent biological replicates and error bars represent the SEM. **B)** Scatter plots of flow cytometry analysis of cell death induction after ganetespib (0, 10 or 25 nM) combined with PRRT or EBRT in GOT1 (left panel) and BON1-SSTR2 (right panel) cells. Forward scatter is shown on the y-axis and SYTOC AADvanced dead cell stain on the x-axis. Gating strategy for live and dead cell populations was performed as indicated.


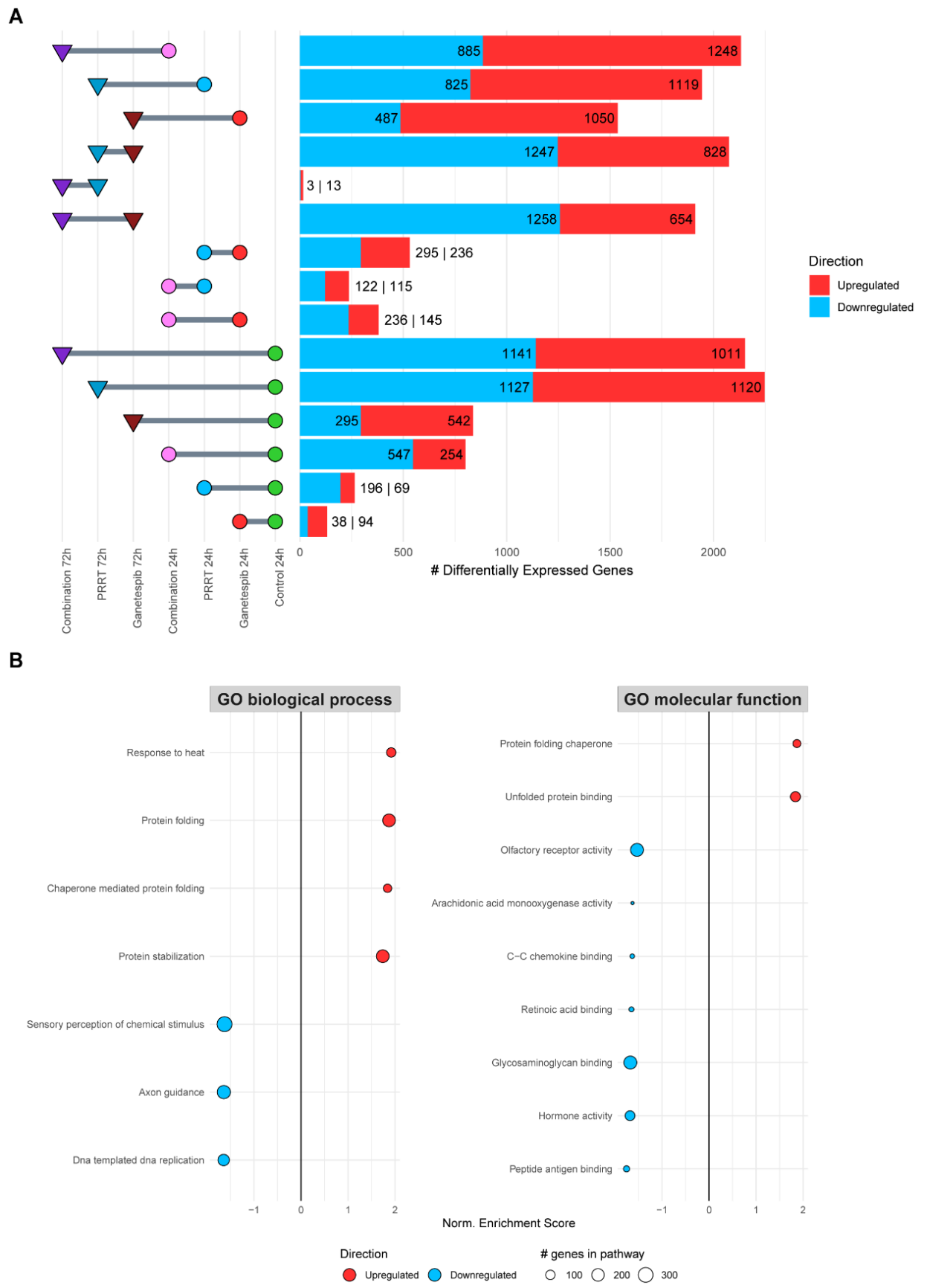


**Supplementary Figure 2. Transcriptomic analysis of the response of GOT1 cells to PRRT and ganetespib mono- and combination therapies. A)** Differential gene expression analysis between groups and conditions after 24 h and 72 h of treatment with PRRT, ganetespib or combination treatment. Additionally, an interaction model was used for the 24 h time point to decouple the effects of PRRT and ganetespib monotherapy. **B)** GSEA results for the comparison of ganetespib and control using GO database after 24 h of treatment. Circle size indicates the number of genes represented in the pathway. Circle color indicates upregulation (red) or downregulation (blue) of the pathway.
